# Polyelectrolyte complexes based on a novel and sustainable hemicellulose-rich lignosulphonate for drug delivery applications

**DOI:** 10.1007/s13346-024-01573-2

**Published:** 2024-03-26

**Authors:** Ioannis Dogaris, Ievgen Pylypchuk, Gunnar Henriksson, Anna Abbadessa

**Affiliations:** 1 Department of Fiber and Polymer Technology, School of Engineering Sciences in Chemistry, Biotechnology, and Health, Royal Institute of Technology, Teknikringen 56-58, Stockholm, SE-100 44 Sweden; 2 Present Address: Department of Materials and Environmental Chemistry, Stockholm University, Svante Arrhenius väg 16C, Stockholm, 10691 Sweden; 3 Center for Research in Molecular Medicine and Chronic Diseases (CiMUS), IDIS Research Institute, Universidade de Santiago de Compostela, Avenida Barcelona s/n, Santiago de Compostela, 15782 Spain; 4 Department of Pharmacology, Pharmacy and Pharmaceutical Technology, School of Pharmacy, Universidade de Santiago de Compostela, Campus Vida, Santiago de Compostela, Spain

**Keywords:** Polyethylenimine, Chitosan, Lignin, Polyelectrolyte complexation, Controlled drug delivery, Sustainability

## Abstract

Polyelectrolyte complexes (PECs) are polymeric structures formed by the self-assembly of oppositely charged polymers. Novel biomaterials based on PECs are currently under investigation as drug delivery systems, among other applications. This strategy leverages the ability of PECs to entrap drugs under mild conditions and control their release. In this study, we combined a novel and sustainably produced hemicellulose-rich lignosulphonate polymer (EH, negatively charged) with polyethyleneimine (PEI) or chitosan (CH, positively charged) and agar for the development of drug-releasing PECs. A preliminary screening demonstrated the effect of several parameters (polyelectrolyte ratio, temperature, and type of polycation) on PECs formation. From this, selected formulations were further characterized in terms of thermal properties, surface morphology at the microscale, stability, and ability to load and release methylene blue (MB) as a model drug. EH/PEI complexes had a more pronounced gel-like behaviour compared to the EH/CH complexes. Differential scanning calorimetry (DSC) results supported the establishment of polymeric interactions during complexation. Overall, PECs’ stability was positively affected by low pH, ratios close to 1:1, and the addition of agar. PECs with higher EH content showed a higher MB loading, likely promoted by stronger electrostatic interactions. The EH/CH formulation enriched with agar showed the best sustained release profile of MB during the first 30 h in a pH-dependent environment simulating the gastrointestinal tract. Overall, we defined the conditions to formulate novel PECs based on a sustainable hemicellulose-rich lignosulphonate for potential applications in drug delivery, which promotes the valuable synergy between sustainability and the biomedical field.

**Graphical abstract:**

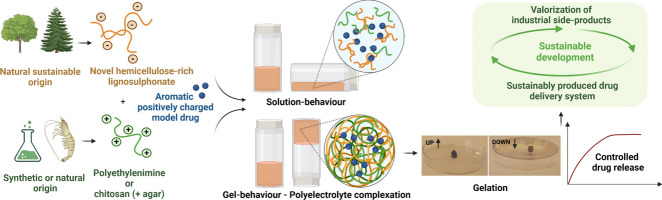

**Supplementary Information:**

The online version contains supplementary material available at 10.1007/s13346-024-01573-2.

## Introduction

Polyelectrolyte complexes (PECs) are polymeric structures formed by the self-assembly of oppositely charged polymers that interact mainly via strong, yet reversible electrostatic interactions [1]. This interpolymer ionic condensation is a spontaneous process that occurs in aqueous media, without the use of chemical solvents or cross-linkers [2]. The driving force for polyelectrolyte complexations is the release of counterions originally associated with the polymers, which makes it an entropy-driven phenomenon [2]. PECs formation can involve the establishment of additional interpolymer interactions, such as dipole interactions, Van der Waals forces, H-bonding and hydrophobic interactions. Several factors affect the formation and stability of PECs, such as charge-to-charge stoichiometry, charge density, molecular weight and concentration of the polyelectrolytes, pH, ionic strength, salt concentration and mode of mixing [3]. Novel biomaterials based on PECs are currently under investigation as protective coatings, smart food packaging materials, surface adhesives and especially as drug delivery systems [4].

The development of PECs for drug delivery applications has received a growing interest since PECs can entrap small-molecule drugs, as well as more fragile therapeutic macromolecules, such as genes, proteins, peptides and antigens [5, 6]. Here, the advantage is that the entrapment occurs under mild conditions and without the establishment of covalent bonds that might cause the inactivation of such therapeutic agents [3]. PECs in drug delivery can facilitate the transport, protect the activity, increase the half-life, control the release kinetics and target a specific release site of their cargos. PECs investigated in drug delivery and tissue engineering applications can give origin to different types of formulations, such as nano- or microparticles, hydrogels, scaffolds, multi-layer films, capsules and tablets [3]. Trends in the field elucidate that natural polymers (especially charged polysaccharides) are usually preferred over synthetic polyelectrolytes, due to their biodegradability, safety, low production costs and natural abundance [2].

Lignin is an aromatic biopolymer made of phenylpropane units and it is a main structural component of plants (15–40% of the dry mass of lignocellulosic biomass) [7]. It has crucial biological roles by preventing excessive swelling of cell walls in water, facilitating efficient water transport, and imparting rigidity to woody plant tissues. These functions are achieved through the covalent crosslinking of lignin with various cell-wall polysaccharides, particularly hemicelluloses [8]. As a result, lignin naturally occurs as a hybrid molecule with polysaccharide chains attached. Lignin is available in large amounts as a side-product of the pulp and paper industry that produces paper and other cellulose-based materials, as well as the biorefinery industry, which converts biomass into energy and chemicals [7]. This by-product is normally not soluble in water at neutral pH, however, when it originates from a sulphite pulping process (i.e., the process by which wood pulp is produced by treating wood with solutions of sulphite and bisulphite ions), it occurs in the form of water-soluble sulphonated lignin, which is called lignosulphonate [9].

The lignosulphonate derivative produced by the company Ecohelix is a novel hemicellulose-rich type of lignosulphonate, which has been fully characterized by our group and it is further referred to as EH [10]. Unlike classical lignosulphonate, EH has a unique and amphiphilic nature provided by the randomly distributed segments of sulphonated lignin and hemicellulose. Remarkably, the hemicellulose content of EH is 5-times higher than that of lignosulfonate and its molecular weight is 3-times higher than that of lignosulfonate, which makes EH a much more promising lignosulphonate derivative for material science and high-value lignin applications [10]. EH precursors are extracted in a sustainable way from the side stream of a sulfite pulping process and are further cross-linked by enzymatic treatment to yield a high molecular weight lignosulphonate-hemicellulose hybrid polymer that is technically considered bio-based to an extent of 97%. The presence of sulfonic acid groups and phenolic groups on the lignin segments, as well as the carboxyl groups on the hemicellulose chains, make EH a strong polyelectrolyte ideal for the formation of PECs. This feature has been previously exploited by our group for the development of multi-layer coatings for food packaging [11].

Importantly, the presence of sulfonic acid groups makes EH a negatively charged polymer at all possible pH values, while its charge density increases by increasing the pH due to the progressive deprotonation of the other ionizable groups with higher pK_a_. Hence, EH can interact with positively charged polyelectrolytes and drug molecules at any possible pH. Remarkably, the presence of aromatic rings in the lignin backbone makes EH a suitable polymer for the entrapment of therapeutic compounds with aromatic structure, via π-π stacking interactions. These are non-covalent attractive interactions that take place between the π bonds of aromatic rings during orbital overlap and are currently investigated for the entrapment and controlled release of aromatic drugs, such as paclitaxel and docetaxel [12–15]. In addition to ionic and π-π stacking interactions, other non-covalent forces, such as H-bonds, may strengthen EH interactions with therapeutic molecules [16].

The aim of this study was to investigate the formation of PECs made by combining the novel sustainable polymer EH with two positively charged polyelectrolytes, namely polyethylenimine (PEI) and chitosan (CH) for the entrapment and controlled release of a model drug, i.e., methylene blue. PEI was selected because it has been previously shown to form complexes with lignosulphonate for material science applications [17] and because it is a polycation widely investigated in the drug delivery field [18–20]. Indeed, it is one of the most studied polymers due to its high capacity to complex DNA in the so-called polyplexes, which are currently the most promising alternative to viral vectors for gene delivery [21]. Moreover, being PEI a synthetic polymer, it has the advantage of being produced with different molecular weights and structural forms (linear vs. branched) in a reproducible way. This allows the fabrication of pharmaceutical formulations with tunable features. CH was chosen because it is widely investigated for the preparation of PECs-based nano-/microparticles, beads, hydrogels, films and membranes in the drug delivery field [1–3, 22, 23]. Chitosan participation in the complexation process relies on the protonation of its amino groups in an acidic medium, which makes it a strong polyelectrolyte able to interact with a large variety of polymers and proteins that show a net negative charge at acidic pH, the only condition where chitosan is water-soluble [22].

In this study, we investigated the effect of polyanion/polycation ratio, type of polycation, and working temperature on the formation of stable PECs in the form of hydrogels. We further characterized the prepared PECs in terms of thermal properties, surface morphology, and stability under different conditions. Finally, we studied the potential use of these PECs for the entrapment and the gastrointestinal controlled release of an aromatic, positively charged model drug, namely methylene blue. The novelty of this study involves the promising combination of the sustainability field with the biomedical field, by making use of a novel and sustainable lignosulphonate-hemicellulose polymer for drug delivery applications. Current literature on the topic reveals that the use of lignin for biomedical applications is motivated by its unique physico-chemical properties, as well as by its antioxidant and anti-microbial activity [7]. The exploitation of these properties in drug delivery and tissue engineering is a novel and promising approach that is gaining a growing interest in the scientific community, as demonstrated by the increasing number of publications in the last decade [7, 24]. On one hand, this interdisciplinary approach will allow the valorization of modern pulp mills and biorefineries by the development of high-value products from an inexpensive side-product. On the other hand, it will provide pharmaceutical researchers with a novel, biodegradable and sustainable polymer with unique physico-chemical properties, as a valid alternative to fossil-based raw materials. This will be a key action for the implementation of bioeconomy strategies aiming at a sustainable modern development of the biomedical field.

## Materials and methods

### Materials

**Fig. 1:**
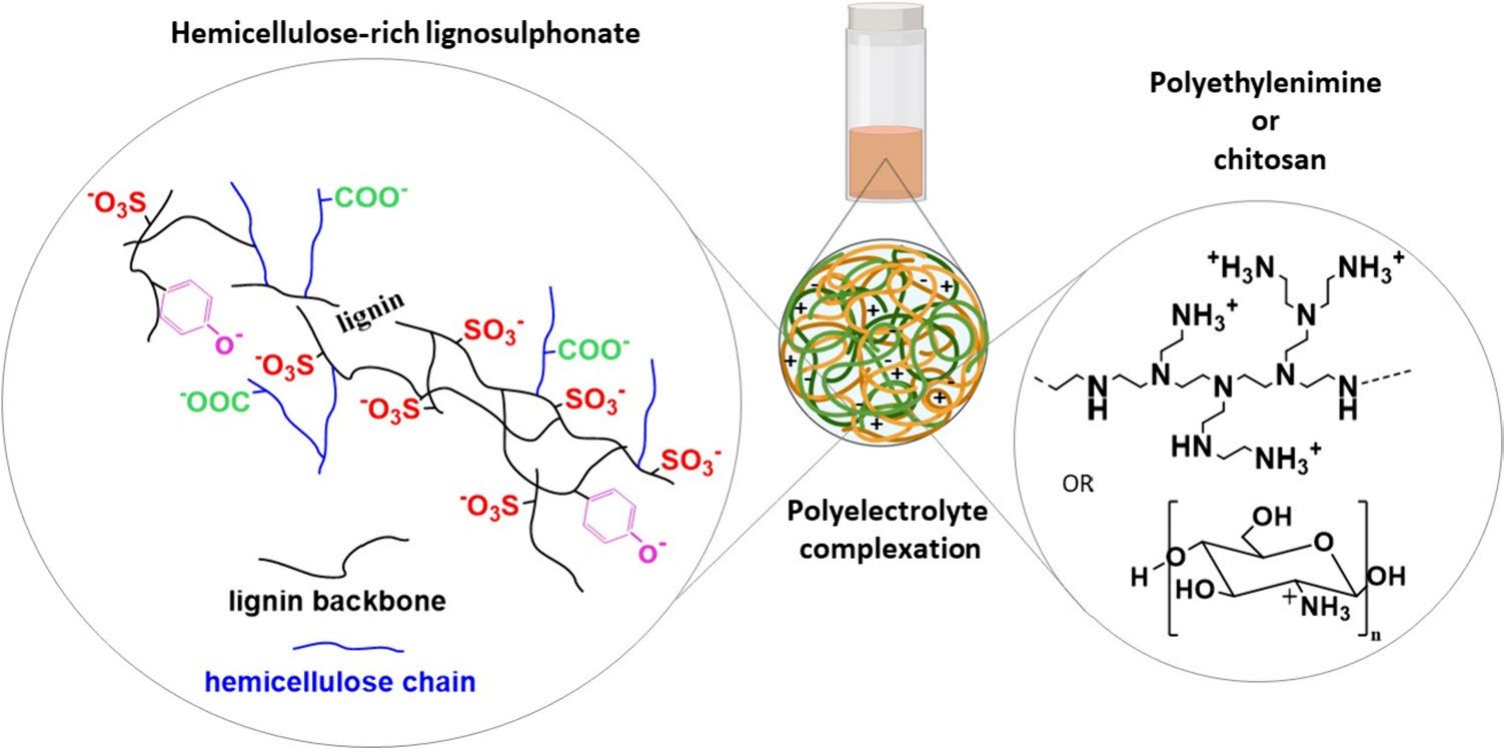
Scheme of polyelectrolyte complexation between the Ecohelix hemicellulose-rich lignosulphonate polymer (simplified representation of chemical structure, left side) and polyethylenimine or chitosan (chemical structures, right side)

Ecohelix lignosulphonate-hemicellulose hybrid polymer (EH, schematic composition shown in Fig. 1) was kindly donated by Ecohelix AB (Stockholm, Sweden) as an aqueous solution (approximately 26% w/w). After freeze-drying, the obtained powder was stored at room temperature until further use. A full characterization in terms of chemical composition, thermal properties and molecular weight of EH has been previously reported by our group [10]. Branched polyethyleneimine (PEI, aqueous solution 50% w/w, Mw = 60 000 Da, chemical structure reported in Fig. 1) was supplied by Acros Organics. Chitosan (CH, Mw = 310 000–375 000 Da, deacetylation degree > 75%, chemical structure reported in Fig. 1) was purchased from Sigma Aldrich (Stockholm, Sweden). Agar (“agar-agar for microbiology”) was purchased from Merck (Darmstadt, Germany) and abbreviated herein as AG. Methylene blue dye (MB) was supplied by Sigma Aldrich (Stockholm, Sweden). All other chemicals were supplied by Sigma Aldrich (Stockholm, Sweden).

### Preparation of polyelectrolyte complexes (PECs) based on EH and PEI or CH

A PEI solution was prepared by dissolving 100 g/L of PEI in Milli-Q water and adjusting the pH to 8.0 using 1 N NaOH. A CH solution was prepared by dissolving 10 g/L of CH in 1% (v/v) acetic acid aqueous solution overnight and adjusting the pH to 5.0 using 1 N NaOH. Two EH solutions were prepared in Milli-Q water to match the concentrations and pH values of the PEI and CH solutions: (a) 100 g/L, pH 8.0 and (b) 10 g/L, pH 5.0. NaCl (0.01 M) was added in all polymer solutions to a total volume of 0.75-1 mL. Initially, various PECs were screened by mixing EH with PEI or CH at different ratios (i.e., 1:3, 1:2, 1:1, 2:1, 3:1, 5:1, and 9:1, by weight) and incubating them at different temperatures (i.e., -20, 4, 23, 50, and 90 °C) for 30 min (*n* = 2). For PECs treated at -20 °C, starting solutions were quickly mixed at room temperature, subsequently frozen at -20 °C, and finally thawed to room temperature. A general scheme of polyelectrolyte complexation and chemical structures of polyelectrolytes is reported in Fig. 1. AG (5, 10, 15 and 20 g/L) was added to the EH solution as a thickening agent to improve complexation with CH. This first screening allowed the selection of four PECs prototypes that were further investigated in follow-up experiments and prepared fresh each time using these recipes: (1) EH:PEI 2:1, 0.25 mL PEI 100 g/L added in 0.5 mL EH 100 g/L, incubated for 30 min at 23 °C; (2) EH:PEI 9:1, 0.1 mL PEI 100 g/L added in 0.9 mL EH 100 g/L, incubated for 30 min at 90 °C; (3) EH:CH 1:2, 0.5 mL CH 10 g/L added in 0.25 mL EH 10 g/L, incubated for 30 min at 90 °C; (4) EH:CH:AG 1:1:2, 0.5 mL CH 10 g/L added in 0.5 mL EH 10 g/L with 20 g/L agar, incubated for 30 min at 90 °C.

### Qualitative assessment of gelation and injectability

 A selected PECs prototype with gel-like behaviour, i.e., EH/PEI 1:1 (at 23 °C) was qualitatively assessed to visualize hydrogel formation, flow under standard gravitational force and injectability. To this aim, PECs were prepared as described in Section “Preparation of polyelectrolyte complexes (PECs) based on EH and PEI or CH” in a glass vial. Subsequently, the vial was turned upside-down to visualize the gel stability under the gravitational force. A hydrogel volume of 100 µL was taken up with a positive displacement pipette and drop-casted on a plastic Petri dish. Finally, the Petri dish containing the hydrogel drop was turned upside-down to visualize the hydrogel stability immediately after injection.

### Differential scanning calorimetry (DSC)

PECs prepared as described in Section “Preparation of polyelectrolyte complexes (PECs) based on EH and PEI or CH” were dried at room temperature for 1 week. Samples with a mass of 2–5 mg were placed in 40-µL aluminum crucibles and heated from 30 to 625 °C at a rate of 10 °C/min, under a N_2_ flow rate of 50 mL/min, using a Mettler-Toledo DSC 820 instrument.

### Scanning electron microscopy (SEM)

Freshly prepared PECs were drop-casted over a silicon wafer and air-dried for 1 week. Samples were sputter-coated with a 2 nm layer of Pt-Pd layer using a Cressington 208 h high-resolution coater (Cressington Scientific Instruments, UK) and further visualized using a field-emission scanning electron microscope (FE-SEM) S-4800 (Hitachi, Japan).

### Stability of PECs

Selected PECs (EH:PEI 2:1 at 23 °C, EH:PEI 9:1 at 90 °C, EH:CH 1:2 at 90 °C, and EH:CH:AG 1:1:2 at 90 °C) were prepared as described above in 15-mL centrifuge tubes for each stability test (*n* = 3). Three different media were used in this test, i.e., Milli-Q water and two different solutions to simulate the intestinal and gastric conditions. Phosphate buffered saline (PBS) with a pH 7.4 (NaCl, 8.0 g/L; KCl, 0.2 g/L; Na_2_HPO_4_, 1.44 g/L; KH_2_PO_4_, 0.24 g/L) was used to simulate intestinal fluid, whereas a solution with a pH 1.2 (NaCl, 2.0 g/L; HCl, 0.7% v/v) was used to simulate gastric fluid [25]. A volume of 10 mL of either Milli-Q water, PBS or acidic solution was added in each tube and centrifuged at 4000 rpm for 5 min. The supernatant was separated to quantify the leached EH, while the remaining material was dried first in the air for 24 h and then in an oven at 60 °C until constant weight to estimate its dry weight. From this, the weight loss (%) of each sample in each solution was calculated. The EH concentration in each supernatant was determined by measuring the UV absorbance (*A*_280_) using a UV-vis spectrophotometer (Shimadzu, UV2550) and a calibration curve based on EH solutions with known concentrations (0.01–0.1 mg/mL), as previously described for lignosulphonate [26]. The values of EH leaching during the first wash of PECs in each fluid were reported, since only minimal losses were observed during the second wash, which were virtually nil after the third wash.

An additional set of triplicates of each of the selected PECs prototypes was prepared in parallel in 15-mL tubes without any extra media added for dry weight estimation after initial complexation as a basis for the weight loss estimation during the stability tests. Briefly, each PEC-containing tube was centrifuged (4000 rpm for 5 min), and after discarding the supernatant (non-complexed polymers and leaking water) the remaining material was air-dried for 24 h and then oven-dried at 60 °C until constant weight to estimate the average dry weight of each PECs prototype.

### Loading of MB in PECs

To facilitate drug loading, MB (5 g/L) was dissolved in the stock EH solutions before complexation as follows: (a) 50 mg of MB in 10 mL EH 10 g/L pH 5 (MB:EH ratio 1:2); (b) 50 mg of MB in 10 mL EH 100 g/L pH 8 (MB:EH ratio 1:20); (c) 50 mg of MB and 20 mg AG in 10 mL EH 10 g/L pH 5 (MB:EH ratio 1:2). Selected PECs were prepared in 50-mL centrifuge tubes (*n* = 3) as described above. The MB loading efficiency (LE) and percent loading (L) were estimated using an indirect method. To this aim, after preparation, each PECs prototype was left to stand for 24 h at room temperature, then 30 mL of PBS pH 7.4 was added, and finally the tubes were centrifuged at 1000 rpm for 5 min. The supernatant was collected to estimate the loaded amount of MB by subtracting the dissolved MB (mg) from the MB amount (mg) in the initial loading solution. The amount of the loaded MB was normalized based on the average dry weight of each PECs prototype. The loading efficiency (*LE*) was calculated according to Eq. (1):

1 $$LE \left(\%\right)=\frac{{M}_{load}}{{M}_{0}}\times 100\%=\frac{({M}_{0}-{M}_{lost})}{{M}_{0}}\times 100\%$$

where *M*_load_ is the amount (mg) of MB loaded in the PECs, *M*_0_ is the initial MB amount (mg) in the loading solution, and *M*_*lost*_ is the amount (mg) of MB lost (released) in the supernatant. Percent loading (L) was calculated according to Eq. (2):

2 $$L \left(\%\right)=\frac{{M}_{load}}{{M}_{PECs}}\times 100\%$$

where *M*_load_ is the amount (mg) of MB loaded in the PECs and *M*_PECs_ is the dry mass of loaded PECs (mg).

MB quantification in the supernatant was carried out by using a UV-vis spectrophotometer (Shimadzu, UV2550), and by measuring the MB absorbance at 663 nm [27]. Concentrations of MB were obtained using a standard curve based on solutions of known amounts of MB (1–10 mg/L) in 10 mM NaCl. Finally, the tubes containing the PECs were used for the release study as described in the following section.

### In vitro release of MB from PECs

A volume of 30 mL of the acidic solution at pH 1.2 (simulated gastric fluid) was added in each tube containing PECs. The tubes were closed and incubated for 3.5 h at 37 °C in a shaker at 100 rpm. After 3.5 h, the tubes were centrifuged at 1000 rpm for 5 min, the supernatant was decanted, and 30 mL of PBS pH 7.4 (simulated intestinal fluid) was added in each tube. The tubes were then incubated in a shaking (100 rpm) incubator at 37 °C for 30 h, which is considered the minimum retention time in the intestinal tract [25]. A sample of 1 mL was drawn at specific time points during both stages to quantify the released MB. The concentration of MB in each release sample was determined using the UV-vis spectroscopy method described above for the determination of the LE (%) and L (%). The amount of MB released over time was normalized based on the previously estimated average dry weight of each PEC prototype and expressed in mg of released MB per grams of material dry weight. Released MB amounts were plot as a function of time in cumulative and distributive release curves. Furthermore, cumulative release was also plot as a function of the square root of time, and a simple linear regression analysis was carried out using the GraphPad Prism 9.0.0 software.

## Results and discussion

### Effect of polyelectrolyte ratio and temperature on complexation

The rationale behind this study lies in the hypothesis that PECs can be formed by the electrostatic interactions between the negatively charged EH and the positively charged PEI or CH, and that these PECs can be exploited as drug delivery systems. An explorative screening was initially conducted to understand the effect of the polyelectrolyte ratio and temperature on the complexation process, and to identify the conditions for the fabrication of insoluble PECs with a macroscopically gel-like or solid-like behaviour. For the EH/PEI complexation, the working solutions were pH-adjusted to 8, to maximize EH charge density. In fact, EH, as other lignosulphonates polymers, is negatively charged at all practical pH values due to the presence of the sulphonate groups, but importantly, its charge density increases by increasing the pH due to the progressive deprotonation of carboxyl groups (pK_a_ ca. 2.7) and phenolic hydroxyl groups (pK_a_ = 6–11) [10, 11, 26]. This working pH value of 8 could not be used for the EH/CH complexation, due to the fact that CH is only soluble below its pK_a_ of ca. 6.5. For this reason, EH/CH complexation was carried out at pH 5, a condition under which both polymers are soluble and charged [10, 11, 26].

The effect of the polyelectrolytes ratio (EH:PEI and EH:CH ratios from 1:3 up to 9:1) and temperature (from − 20 **°**C up to 90 **°**C) was studied, and the visual outcomes (liquid-, gel-, or solid-like behaviour of the polymeric mixtures) are summarized in Table 1.

**Table 1:** Effect of polyelectrolytes ratio and temperature on the formation of EH/PEI and EH/CH complexes. Prototypes selected for follow up experiments are shown with a bold, underlined symbol

	**EH/PEI complexes**								**EH/CH complexes**							
**Ratio**	**EH**	1	1	1	2	3	5	9	**EH**	1	1	1	2	3	5	9
	**PEI**	3	2	1	1	1	1	1	**CH**	3	2	1	1	1	1	1
**Temperature**	-20 °C*	+	++	++	+	+	-	-	-20 °C*	-	-	-	-	-	-	-
	**4 °C**	-	+	++	++	+	-	-	**4 °C**	-	-	-	-	-	-	-
	**23 °C**	-	+	**+**^**§**^	**++**	+	-	-	**23 °C**	-	-	-	-	-	-	-
	**50 °C**	+	+	+	+	+	-	-	**50 °C**	-	-	-	-	-	-	-
	**90 °C**	+	+	+	+	+	+	**+**	**90 °C**	+	**+**	**-**^**Ω**^	-	-	-	-

* Observation after thawing to room temperature

- Liquid-like behaviour (polymers remain in solution)

+ Gel-like behaviour (possible handling with a positive displacement pipette as described in Section “Qualitative assessment of gelation and injectability”)

++ Solid-like behaviour (a compact solid mixture is observed that can be handled by a spatula)

^§^ selected for the qualitative assessment of gelation (Section “Qualitative assessment of gelation and injectability”)

^Ω^ selected after agar addition at a EH:CH:AG ratio of 1:1:2

All polymeric mixtures presented a brown appearance due to the presence of EH, which is a brown polymer due to the high content of lignin [10]. Overall, EH seemed to form more stable complexes with PEI compared to CH. Indeed, the majority of EH/PEI mixtures presented a gel-like or solid-like behaviour, except for those prepared with EH:PEI ratios of 1:3 (at 4 and 23 °C), 5:1 and 9:1 (at all temperatures < 90 **°**C), which presented a liquid-like behaviour, likely due to the unbalanced charge stoichiometry which triggered macro-phase separation. This is the separation of the system in distinct phases on a large, macroscopic scale, characterized by regions with predominantly polyanionic (EH-rich domains) or polycationic (PEI-rich domains) character, leading to a system with lost structural integrity and a liquid-like behaviour, due to the lack of homogeneously distributed electrostatic interactions. The charge density of EH and PEI at pH 8 is 2.2 meq/g and 4 meq/g, respectively. It follows that a EH:PEI mass ratio of approximately 2:1 is necessary to achieve a charge stoichiometry of 1:1, which is the ratio under which insoluble PECs are usually formed [26]. This explains why mixtures prepared with a EH/PEI ratio close to 2:1 had a better macroscopic outcome at any temperature (gel-like or solid-like behaviour, indicated in the table with + and ++, respectively) compared to mixtures prepared with more unbalanced ratios. Overall, when using these unbalanced EH:PEI ratios (i.e., 1:3, 5:1 and 9:1), the increase of temperature resulted in the formation of homogeneous gel-like mixtures, likely because at higher temperatures the mobility of the polymeric chains increases, allowing a better mixing and limiting the macro-phase separation. It needs to be noted that for samples treated at –20 **°**C, starting solutions were quickly mixed at room temperature, subsequently frozen at –20 **°**C, and finally thawed to room temperature for the evaluation of the gel-like behaviour. It follows that the samples likely underwent a partial complexation at room temperature, which was subsequently slowed down or temporarily stopped during freezing and finally reactivated during thawing. Mixtures with a EH:PEI ratio of 2:1 (at 23 **°**C) and of 9:1 (at 90 **°**C) were selected for follow up experiments. The first one was chosen because it was the only solid-like mixture among those prepared at the most convenient temperature of 23 **°**C. The second one was chosen since it was the only mixture with a gel-like behaviour among those prepared with the highest EH:PEI ratio, which is considered beneficial to obtain a high loading of aromatic, positively charged drugs, such as methylene blue tested in this study.

In contrast to EH/PEI mixtures, the majority of EH/CH mixtures presented a liquid-like behaviour at almost all tested temperatures, likely due to the much lower polymer concentration used, which in turn was dictated by the lower CH solubility, compared to that of PEI. Only when operating at 90 °C, gel formation was visible for mixtures with EH:CH ratios of 1:3 and 1:2. This finding is in line with the results reported by Fredheim et al., who observed compact precipitates during complexation of chitosan and lignosulfonate only at 100 °C, whereas at 50 and 4 °C the complexation resulted in fluffy aggregates [26]. This highlights that the complexation process is entropy-driven, as usually found for polyanion/polycation complexation, where an increase in entropy due to the release of counter-ions associated with the polymers drives the establishment of polymer-polymer electrostatic interactions [26, 28]. To facilitate the formation of more stable, gel-like EH/CH complexes, the addition of AG as a thickening agent was studied. AG is a galactose-based polysaccharide extracted from red algae and it is composed of agarose and agaropectin, usually in a 70/30 ratio [29]. AG is a negatively charged polysaccharide due to the presence of sulphate and pyruvate groups in agaropectin, and it is currently studied for the formation of hydrogels [30]. The most stable complex was observed when using an agar concentration of 20 g/L, which corresponds to a EH:CH:AG ratio of 1:1:2. Therefore, this prototype was selected for follow up experiments, together with the mixture having a EH:CH ratio of 1:2, which was the mixture with the highest EH content that presented a gel-like behaviour.

In summary, the selected prototypes were those made with the following EH/PEI or EH/CH ratios under the specified temperature conditions: (i) EH:PEI 2:1 at 23 °C, further referred to as EP 2:1; (ii) EH:PEI 9:1 at 90 °C, further referred to as EP 9:1, (iii) EH:CH 1:2 at 90 °C, further referred to as EC 1:2; (iv) EH:CH:AG 1:1:2 at 90 °C, further referred to as EC 1:1 + A. Finally, as a proof-of-concept, the mixture with a EH:PEI ratio of 1:1 at 23 °C was selected for the qualitative assessment of gelation and injectability, as described in the next section.

### Qualitative assessment of gelation and injectability

**Fig. 2:**
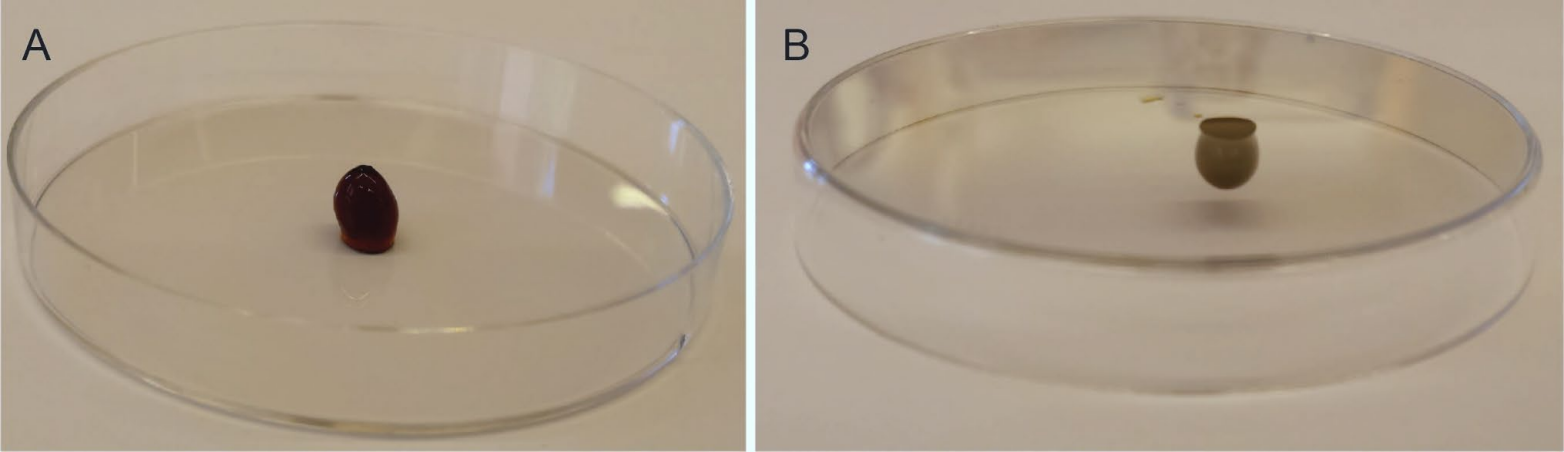
Photographs of a hydrogel made of a mixture with a EH:PEI ratio of 1:1. Shape-stable drop, generated after injecting 100 µL of hydrogel on a Petri dish (A). Shape stability is maintained also after turning the Petri dish upside-down

Gelation and injectability of a mixture with a EH:PEI ratio of 1:1 at 23 °C was qualitatively assessed, as its behaviour is representative of all other mixtures of the study with a gel-like behaviour (marked with + in Table 1). The mixture had a brown, gelling appearance and did not flow under the action of the gravitational force when the vial was turned upside-down, which confirms the formation of a stable hydrogel network. The hydrogel was easily taken up using a positive displacement pipette and, after releasing it on a plastic Petri dish, it retained a drop-like three-dimensional shape (Fig. 2A). High shape fidelity of the generated drop was maintained also after turning the Petri dish upside-down (Fig. 2B). This observation indicates that the hydrogel has a shear-thinning behaviour, i.e., it is able to flow through the tip of a pipette under the applied shear due to the temporary break-down of the physical network. Importantly, the maintenance of the three-dimensional shape after injection demonstrates that the physical network is quickly re-established when the shear stress is removed (recovery phase) [31, 32]. This feature has a high interest in the biomedical field since hydrogels with shear-thinning/fast recovery behaviour are widely investigated as injectable and 3D printable biomaterials in drug delivery and tissue engineering [33–35].

### Thermal properties

**Fig. 3:**
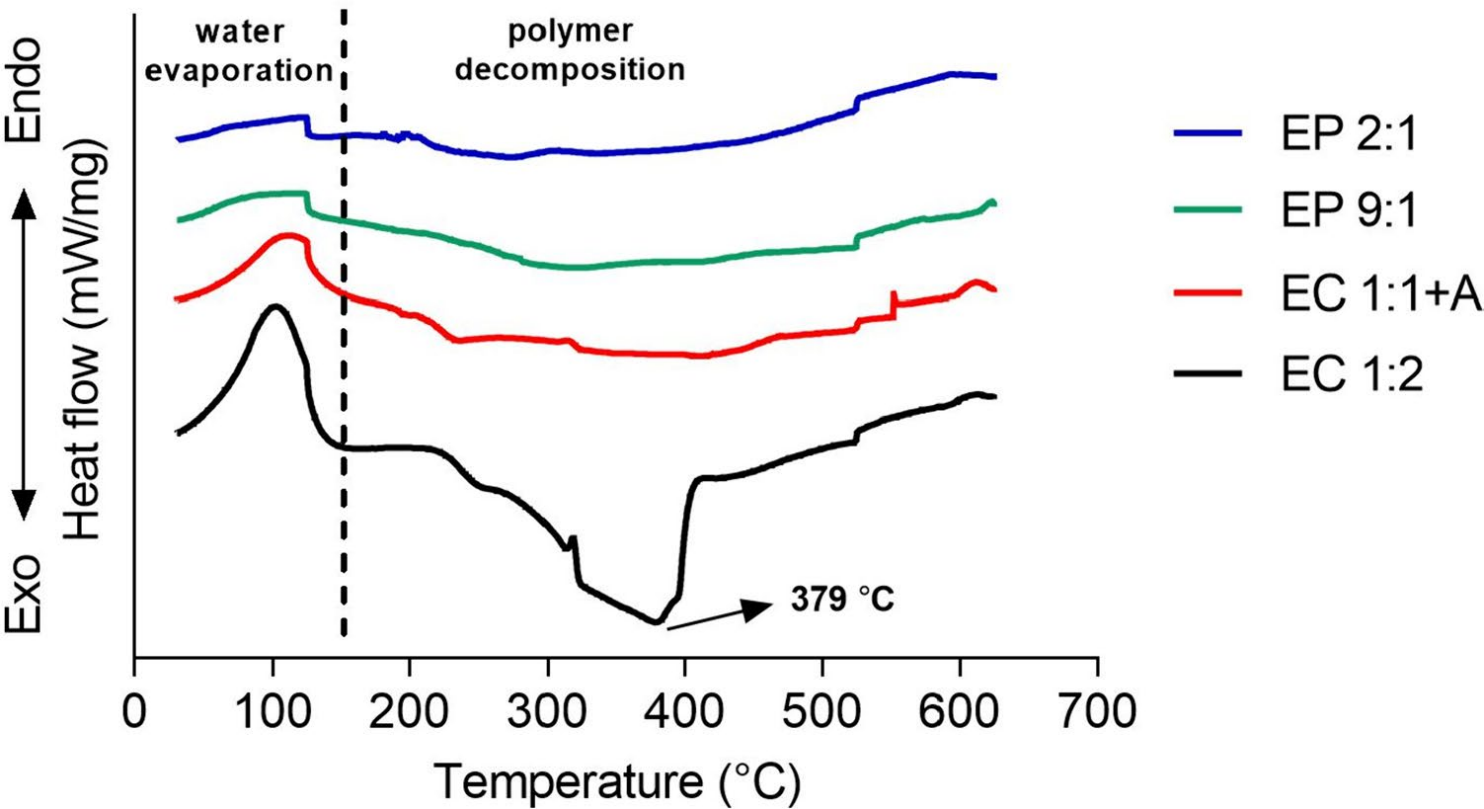
DSC curves over the entire temperature ramp (30–625 °C) for four selected PECs prototypes made of a EH:CH ratio of 1:2 (EC 1:2), a EH:CH:AG ratio of 1:1:2 (EC 1:1 + A), a EH:PEI ratio of 2:1 (EP 2:1), and a EH:PEI ratio of 9:1 (EP 9:1)

DSC analysis is a valuable tool for gaining knowledge on polymer interactions within PECs, by studying, for example, the migration of thermal decomposition peaks associated with depolymerization and pyrolytic reactions of polyelectrolytes [36]. DSC curves for the selected samples are shown in Fig. 3 (separated DSC curves with numerical intervals on the Y-axis are provided in Fig. S1 in the Supplementary Data). The first thermal effect is an endothermic event in the temperature range of 30–150 °C. EC 1:2 and EC 1:1 + A prototypes showed clear peak temperatures at 101 and 113 °C in this region, respectively, whereas for both PEI-containing samples much broader peaks were observed. This thermal event is attributed to the evaporation of water, as previously observed in DSC thermograms of hydrophilic polymers, such as PEI and CH, that are able to attract a significant amount of water [36]. DSC curves showed a more pronounced water evaporation for the EC groups compared to the EP groups, suggesting that in the presence of chitosan, PECs have a higher moisture content. This is in line with our previous findings on the role of water on multilayers based on EH and CH or EH and PEI through electrostatic interactions [11]. In that study, for each of the systems (EH/CH and EH/PEI), we calculated the total adsorbed mass (polymer and associated water) by Quartz Crystal Microbalance with Dissipation (QCM-D) and the amount of adsorbed polymer (without associated water) by Stagnation Point Adsorption Reflectometry (SPAR). Results demonstrated that EH/CH multilayers showed more adsorbed mass per unit area compared to EH/PEI films as measured by QCM. However, SPAR data indicated that EH/CH multilayers contained a much lower polymer content than EH/PEI, revealing that EH/CH system has a higher capacity to interact with water.

In the temperature range of 200–400 °C, polymer decomposition reactions occur. For all samples, broad exothermal peaks were observed in this region, with a visible peak temperature at 379 °C for EC 1:2 sample. These exothermal peaks are attributed to chitosan degradation, involving saccharide dehydration, depolymerization, and the decomposition of amino groups, as well as to the decomposition of sulphonic groups in EH that occurs with SO_2_ release. Peak temperatures for the decomposition of pure chitosan are usually reported between 279 and 311 °C [36–42], and the decomposition of sulfonic groups in lignosulfonate is reported around 350 °C [43]. Importantly, the higher peak temperature observed for EC 1:2 sample (379 °C) suggests the participation of both polymers in the complexation reaction. The absence of sharp decomposition peaks in the region 200–400 °C for EP 2:1, EP 9:1 and EC 1:1 + A samples, likely indicates that much stronger polymer interactions were present in these samples compared to EC 1:2 sample.

### Surface morphology

**Fig. 4:**
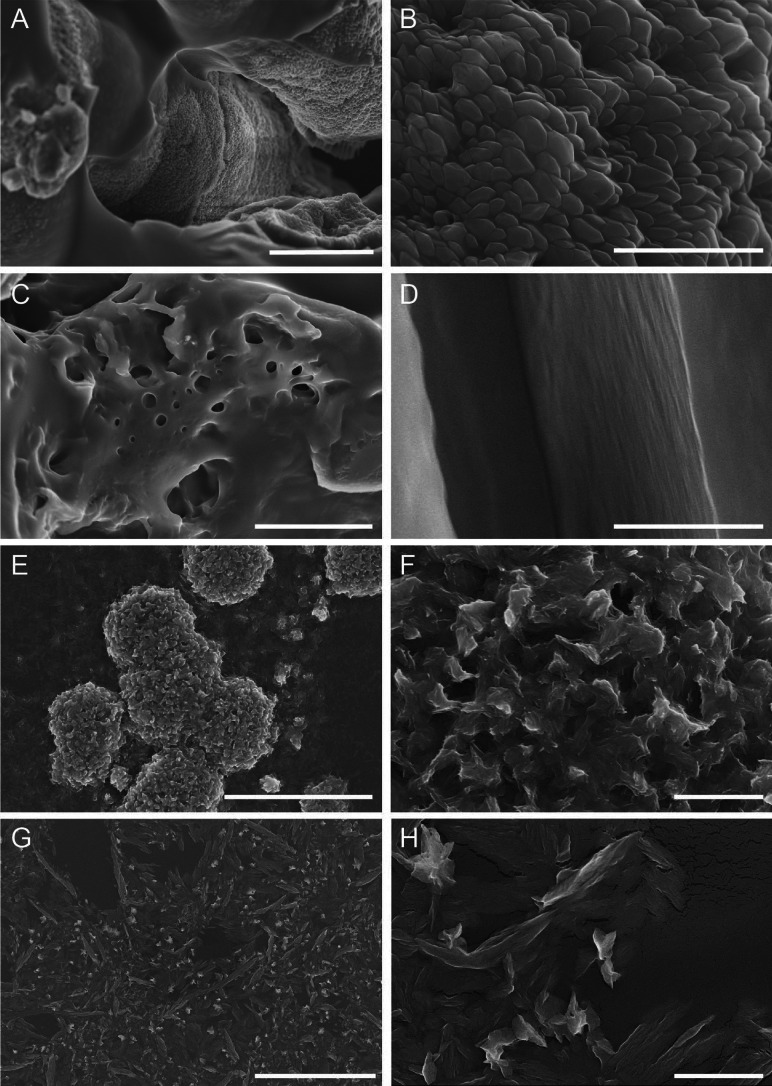
SEM photographs of dried PECs made of EH and PEI, i.e., EP 2:1 (**A**, **B**) and EP 9:1 (**C**, **D**); and made of EH and CH, i.e., EC 1:2 (**E**, **F**) and EC 1:1 + A (**G**, **H**). Scale bar indicates 10 μm in **A**, **C**, **E**, **G** and 1 μm in **B**, **D**, **F**, **H**

SEM photographs for selected PECs made of EH and PEI or CH are presented in Fig. 4. PECs made of EH and PEI, i.e., EP 2:1 and EP 9:1 are shown in Fig. 4A-D. The matrix of EP 2:1 sample was composed of relatively homogeneous polyhedral-shaped spikes, having a face length of approximately 100 nm. On the other hand, EP 9:1 sample did not have any well-defined microscopic structure, likely due to the large excess of EH with respect to PEI.

PECs composed of EH and CH assembled in the form of micro-sized flakes. More specifically, EC 1:2 (Fig. 4E-F) formed flakes of irregular shape, which fused into large microparticles with a diameter of approximately 5 μm. In contrast, flakes in the EC 1:1 + A sample (Fig. 4G-H) were organized into elongated microstructures with a length of approximately 1–10 μm. The morphological differences between the EC 1:2 and EC 1:1 + A samples are mainly attributed to the addition of agar, which facilitates the assembly of CH:EH flakes into elongated structures. Likely, CH:EH flakes assemble along the agar molecules, forming long microfibrils and an overall more homogeneous matrix compared to that of the agar-free sample, i.e., EC 1:2.

### Stability of PECs

The stability data of the four selected PECs in pure water, acidic solution (pH 1.2) and PBS (pH 7.4) are summarized in Table 2. EP 2:1 was the most stable complex in all three fluids, exhibiting the lowest lignin leaching (3.1–8.3%) and weight loss (≤ 8.5%) compared to the other samples of the study. This minimal EH leaching is explained by the fact that the EH:PEI mass ratio of 2:1 used in this sample corresponds approximately to a 1:1 charge stoichiometry, which is the ratio under which insoluble PECs are usually formed, as explained in Section “Effect of polyelectrolyte ratio and temperature on complexation” In EP 2:1 samples, EH leaching seemed somewhat pH-dependent, showing the lowest value at pH 1.2, likely due to the high protonation degree of PEI at this pH. An increase of the EH:PEI ratio to 9:1 resulted in a 4-11-fold increase in EH leaching, reasonably due to the loss of unbound EH, that was used in large excess.

**Table 2:** Stability and EH leaching of PECs in pure water, at pH 1.2 (simulated gastric fluid) and pH 7.4 (simulated intestinal fluid) (± is the standard deviation, *n* = 3)

**PEC**	**Pure water**		**pH 1.2**		**pH 7.4**	
	**EH leaching %**	**Weight loss %**	**EH leaching %**	**Weight loss %**	**EH leaching %**	**Weight** **loss %**
**EP 2:1**	5.8 ± 0.5	6.5 ± 7.1	3.1 ± 0.9	8.5 ± 3.9	8.3 ± 3.9	-4.2 ± 0.6*
**EP 9:1**	32.3 ± 2.6	33.8 ± 3.9	34.2 ± 5.7	30.5 ± 7.4	30.0 ± 7.4	15.9 ± 7.3
**EC 1:2**	95.6 ± 5.1	69.7 ± 18.0	80.1 ± 4.2	68.9 ± 6.9	95.2 ± 4.4	35.4 ± 4.0
**EC 1:1 + A**	28.4 ± 4.0	71.3 ± 6.0	23.7 ± 1.0	81.4 ± 1.1	27.1 ± 4.0	62.3 ± 0.5

*The slight gain in weight for this sample is attributed to the absorption of PBS salts

Both chitosan-containing complexes (EC 1:2 and EC 1:1 + A) had higher weight loss in all fluids compared to PEI-containing complexes, and the agar-free sample EC 1:2 showed the highest EH leaching of all samples of the study (80.1–95.6%). This confirms the low stability of PECs in the absence of agar due to the low polymer concentration used, as explained in Section “Effect of polyelectrolyte ratio and temperature on complexation”. In contrast, the addition of agar significantly reduced EH leaching (23.7–28.4% for EC 1:1 + A sample), likely due to the increase of the matrix viscosity which reduced EH diffusion. As observed for EP 2:1 sample, also for CH-containing samples, the acidic conditions reduced EH leaching, likely due to the high charge density of chitosan at pH 1.2. Weight loss values for EC 1:1 + A samples under the different pH conditions were higher (62.3–81.4%) compared to those found for EC 1:2 samples (35.4–69.7%), despite the lower EH leaching, which suggests a certain agar release during the washing. For all samples, when comparing weight loss values in PBS with those in water, values in PBS were systematically lower than those in water. This does not directly indicate a higher stability of PECs in PBS, since weight values used for the weight loss calculation refer to the total dry mass including the content of potentially absorbed salts.

Taken altogether, EH leaching depended on pH, PECs composition and matrix viscosity, being low pH, 1:1 charge ratio and high viscosity the most appropriate conditions to limit EH loss. For all pH conditions, the yield followed the trend EP 2:1 > EP 9:1 > EC 1:2 > EC 1:1 + A, indicating that PEI-containing samples were overall more stable than CH-containing samples and that a 1:1 charge stoichiometry positively affects the yield.

### Loading and release of MB

**Fig. 5:**
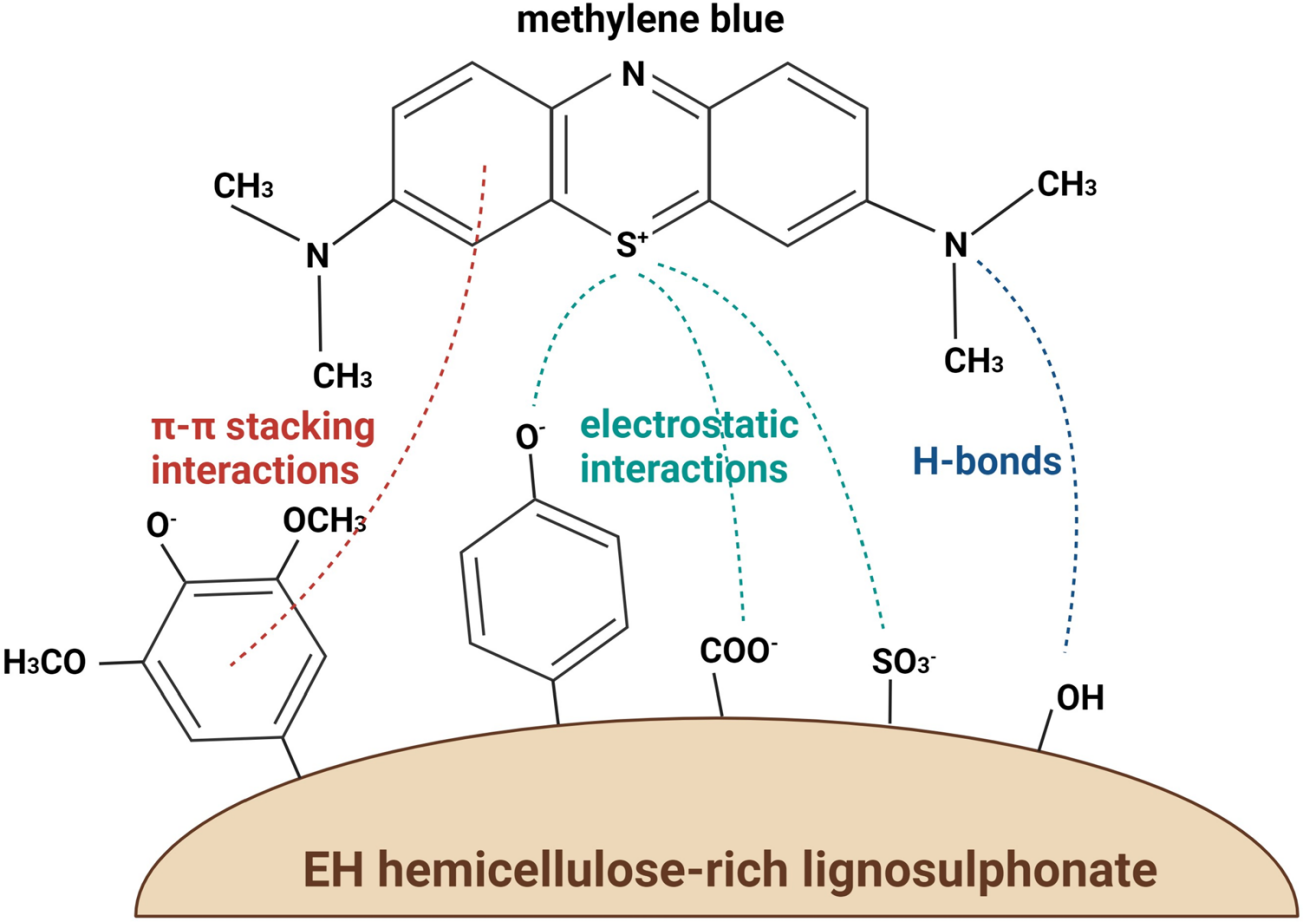
Schematic illustration of some of the possible interactions between methylene blue and EH hemicellulose-rich lignosulphonate

Loading of MB in PECs made of EH and PEI or CH was performed by mixing MB in the EH solution to allow drug-polymer interactions prior to PECs formation. Indeed, MB is a positively charged, polycyclic aromatic compound (Fig. 5) that can interact with EH through several types of interactions. Firstly, the positive charges of MB can interact with the negative groups of EH (i.e., phenolic, carboxyl and sulphonic groups). Secondly, H-bonds can occur between the N atoms of MB and the hydroxyl groups of EH. Finally, π-π stacking interactions can be established between the several aromatic rings of EH and those of MB (Fig. 5) [27]. As a consequence of these interactions, the values of loading efficiency were high for all four formulations (89.6–97.9%). The slightly higher encapsulation efficiency of EC 1:1 + A sample compared to EC 1:2 is likely attributed to the higher amount of negatively charged polymers available for ionic interactions with positively charged MB. Indeed, EC 1:1 + A sample contained a higher amount of EH, as well as a slightly negative agar, which was not present in EC 1:2 sample. Moreover, compared to EC 1:2 sample, EC 1:1 + A sample had a much lower EH leaching which provided a higher amount of EH available for interactions with MB. The MB loading of PECs varied between 3.2 and 14.9%, based on the different PECs compositions (Table 3). Considering that the same amount of MB was used for all formulations during loading and that this amount was almost equally entrapped in terms of efficiency, the difference in the MB loading derives from the fact that different formulations have different final masses of loaded PECs, due to different starting polymer concentrations and different weight losses. In line with this, PEI formulations had lower MB loading values than CH formulations due to their higher starting polymer concentration and lower weight loss. Weight loss data reported in Table 2 correlates well with the MB loading data reported in Table 3, as samples that had the lowest weight loss had the lowest MB loading due to the highest mass of loaded PECs. Weight loss increases with the trend EP 2:1 < EP 9:1 < EC 1:2 < EC 1:1 + A (Table 2), which is the same trend followed by the increase of the MB loading (Table 3).

**Table 3:** Values of loading efficiency (LE%) and percent loading (L %) of MB, calculated according to Eqs. 1 and 2 for PECs composed of EH and PEI or CH (+ agar)

**PEC**	**MB Loading efficiency (%)**	**MB loading (%)**
**EP 2:1**	94.6 ± 2.5	3.2 ± 0.8%
**EP 9:1**	93.1 ± 0.1	7.9 ± 0.1%
**EC 1:2**	89.6 ± 1.0	12.6 ± 1.4%
**EC 1:1 + A**	97.9 ± 0.3	14.9 ± 0.4%

**Fig. 6:**
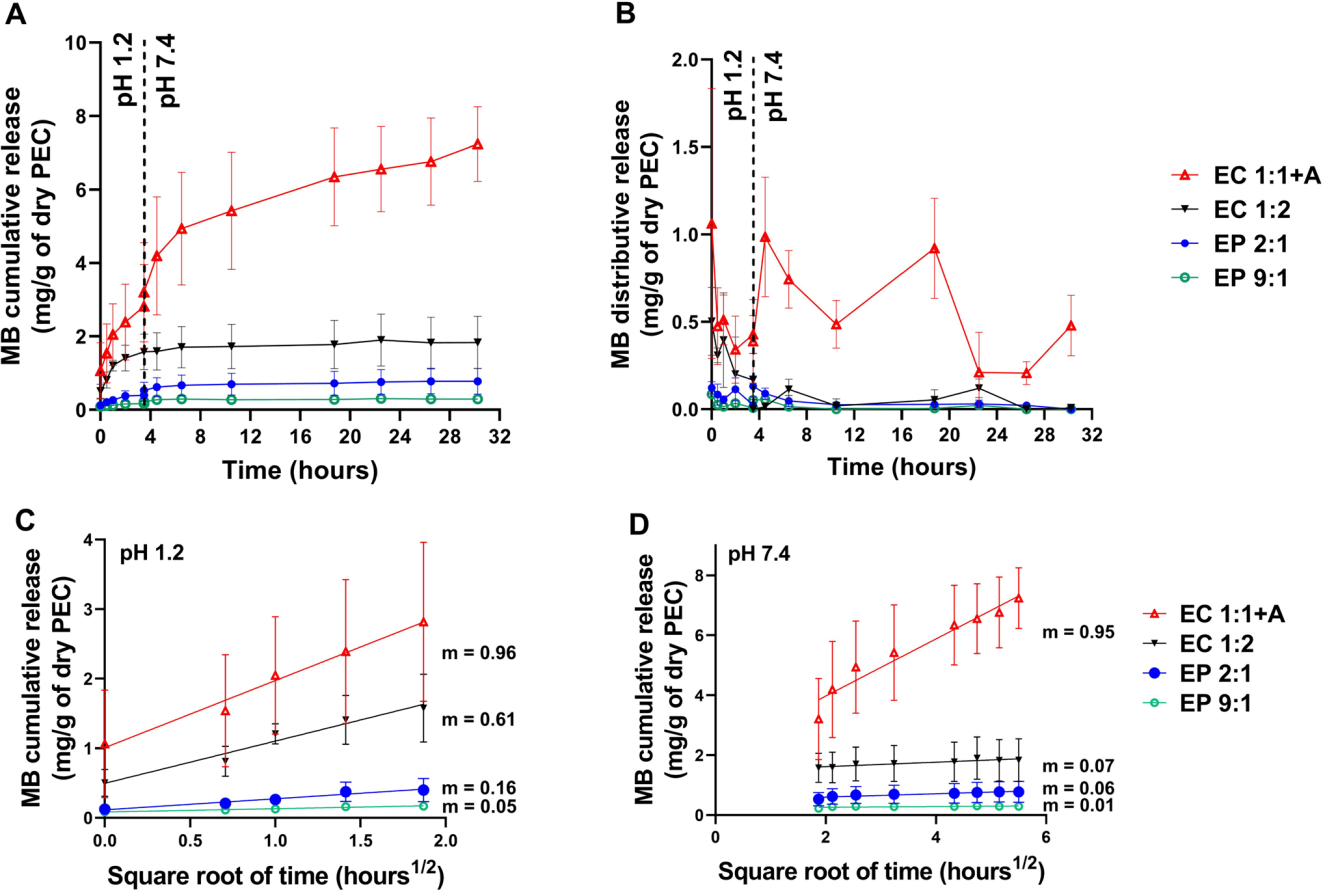
MB release kinetics from PECs. Cumulative (**A**) and distributive (**B**) release of MB (mg/g of dry PEC). Cumulative release as a function of the square root of time at pH 1.2 (**C**) and pH 7.4 (**D**)

Release curves of all MB-loaded PECs during the first 30 h under the two pH conditions tested (simulated gastric and intestinal fluids) are shown in Fig. 6A and B (cumulative and distributive release expressed as mg of released MB out of the dry mass of PECs), whereas curves representing the cumulative release as a function of the square root of time are presented in Fig. 6C (pH 1.2) and 6D (pH 7.4). Cumulative release expressed as % of MB released over the total MB entrapped is reported in Figure S2 (Supplementary Data). Release curves of all agar-free samples were characterized by an initial burst release phase during the first 3.5 h when the gastric transit at pH 1.2 was simulated, followed by a much slower release kinetics that reached a plateau during the subsequent hours when pH was kept at 7.4 (Fig. 6A). In contrast, EC 1:1 + A continuously released MB during the entire time frame. More in detail, for this formulation, two different burst phases were visible, i.e., an initial one at pH 1.2 and a second one corresponding to the pH change (Fig. 6B). During the first 3.5 h, the cumulative release of all PECs scaled linearly with the square root of time, indicating a first-order release kinetics (Fig. 6C). By comparing the slopes of the fitting lines, it is clear that the release rate decreases with the trend EC 1:1 + A > EC 1:2 > EP 2:1 > EP 9:1 (corresponding slope values 0.96, 0.61, 0.16 and 0.05). During the second phase at pH 7.4, all agar-free samples had a very low release rate (Fig. 6D, slope values 0.01–0.07). In contrast, EC 1:1 + A showed a similar release rate to that shown during the acidic phase (Fig. 6D, slope 0.95 vs. 0.96), which highlights the ability of this formulation to release MB in a sustained fashion. Agar is a well-known hydrogel-forming component, and therefore, in EC 1:1 + A sample, it likely contributed to a much higher water uptake compared to the other samples, which in turn facilitated MB diffusion through the polymeric matrix [30]. Remarkably, this sample had the highest MB loading and the greatest amount of MB released over time, which makes it a more promising prototype compared to the others. Taken altogether, in EC 1:1 + A sample, AG acted as a viscosity-enhancer leading to three main effects: (i) acquisition of the gel-like behaviour, (ii) reduction of the EH leaching (Table 2), (iii) increased and sustained MB release. This highlights that AG has an important role. However, it is noted that while AG is needed to boost the release, EH remains a key-component for the PECs formation and the drug retention and loading. In fact, given the chemical structures of AG and EH, as well as the establishment of interactions with the drug, it is reasonable that AG participates in drug retention only to a minor extent compared to EH. Indeed, EH is highly negative and has aromatic rings which are responsible for the retention of positively charged drugs with aromatic rings, like MB, due to the establishment of electrostatic and π-π stacking interactions. In contrast, AG is composed of a mixture of agarose and agaropectin. The first is a neutral linear polymer consisting of repeating units of D-galactose and 3,6-anhydro-L-galactose, whereas the latter is a more heterogeneous and branched fraction of agar, which contains additional substituents, such as negatively charged sulphate and pyruvate groups. However, since agaropectin accounts for only a small fraction of AG, the overall effect is a week negative charge. Given that AG is only slightly negative and does not have aromatic rings in its chemical structure, it can participate in the drug retention only to a minor extent. It can be hypothesized that AG participates in drug retention also by reducing EH leaching and, hence, by providing more EH available for the interactions with the drug. All these observations lead to the conclusion that agar is an important component for the stabilization of PECs and drug release, whereas EH ensures PECs formation and strong interactions with the drug. It follows that the release kinetics is a result of the influence of both components on the entire system. Comparing the two PECs made of EH and PEI, EP 9:1 sample which had the highest loading, resulted in the lowest MB release over time, which indicates that strong interactions are established between MB and the large excess of EH used in this sample. Taken altogether, we defined the conditions to formulate drug-loaded PECs based on a novel hemicellulose-rich lignin and able to control the release of a model drug in the gastrointestinal tract. Our findings are relevant to the current research trends on the topic, since the gastrointestinal controlled release of numerous drugs, which are currently administered intravenously, is still an unmet challenge. An example of this active research subject is the development of novel oral formulations of paclitaxel against colon cancer, where important challenges are related to paclitaxel high lipophilicity, efflux, and affinity for intestinal and liver degrading enzymes [44–49]. Our future studies will focus on the encapsulation in these novel PECs of anticancer drugs such as paclitaxel, which will benefit from π-π stacking interactions similar to those between EH and methylene blue, exploited in this study. In the context of anti-cancer therapy, the high potential of the proposed drug delivery system should also be considered because of the anti-oxidant effect of lignin. Lignin has an intrinsic scavenging effect against reactive oxygen species (ROS), and lignin fibers can adsorb carcinogens and toxins in the colon [50, 51]. This ability has been associated with a reduction of colon cancer relapse that is mainly linked to oxidative stress. This opens up new possibilities for the development of lignin-based drug delivery systems that may synergically improve the effect of chemotherapeutics.

## Conclusions

The supply of sustainable materials to the biomedical field is a crucial aspect to promote valuable synergies between sustainability and the biomedical field, which aim at the replacement of fossil-based raw materials with biomass-derived high-quality materials. On one hand, this approach will provide the biomedical field with novel materials with unique properties. On the other hand, it will contribute to the valorization of industrial side products, such as lignin, for a more circular and sustainable economy. In this study, we combined a novel and sustainably produced hemicellulose-rich lignosulphonate with PEI or CH and agar for the development of drug-releasing PECs. We identified the conditions for stable complexation and demonstrated how the properties of EH-based PECs depend on a variety of parameters, such as polyelectrolyte ratio, type of polycation, temperature and pH. This allowed the identification of the conditions to form EH-based PECs able to provide a sustained release of a model drug in a physiologic environment that simulates the gastrointestinal tract. Future investigations will be oriented toward the use of these PECs for the entrapment and controlled release of hydrophobic drugs and proteins, as well as on further optimization of these PECs in the form of injectable hydrogels for potential applications in tissue engineering. A major focus will be given to the experimental identification of the polymer-drug interactions using a large battery of potential drugs and by combining several analytical techniques, such as UV-VIS spectrophotometry, Fourier Transform Infrared (FTIR) spectroscopy, Zeta potential measurements and X-ray diffraction, among others.

## Electronic supplementary material

Below is the link to the electronic supplementary material.

Supplementary Material 1

## Data Availability

Data will be shared at request after checking compatibility with intellectual property rights.

## Declarations

### Access to images

The graphical abstract and Fig. 5 were created using BioRender.com. Figure 1 was created using ChemDraw Professional, BioRender.com and Microsoft PowerPoint Slide Presentation Software. Figures 3 and 6 were generated using GraphPad Prism 9.0.0.
