# Supplementary material for: Polyelectrolyte complexes based on a novel and sustainable hemicellulose-rich lignosulphonate for drug delivery applications
Source: Drug Deliv Transl Res. 2024 Mar 26;14(12):3452–66. doi: 10.1007/s13346-024-01573-2 (PMC11499397; doi:10.1007/s13346-024-01573-2)
Supplement: Supplementary file 1 — Supplementary Material 1 [file 13346_2024_1573_MOESM1_ESM.docx]

**SUPPLEMENTARY DATA**

**Polyelectrolyte complexes based on a novel and sustainable hemicellulose-rich lignosulphonate for drug delivery applications**

Ioannis Dogaris^1^, Ievgen Pylypchuk^1,2^, Gunnar Henriksson^1^*,* Anna Abbadessa^1,3,4,^***

*^1^ Department of Fiber and Polymer Technology, School of Engineering Sciences in Chemistry, Biotechnology, and Health, Royal Institute of Technology, Teknikringen 56-58, Stockholm, SE-100 44, Sweden.*

*^2^ Present address: Department of Materials and Environmental Chemistry, Stockholm University, Svante Arrhenius väg 16C, Stockholm, 10691, Sweden.*

*^3^* *Center for Research in Molecular Medicine and Chronic Diseases (CiMUS), IDIS Research Institute, Universidade de Santiago de Compostela, Avenida Barcelona s/n, Santiago de Compostela, 15782, Spain.*

*^4^ Department of Pharmacology, Pharmacy and Pharmaceutical Technology, School of Pharmacy, Universidade de Santiago de Compostela, Campus Vida, Santiago de Compostela, Spain.*

*Corresponding author

e-mail address of the corresponding author: [anna.abbadessa@usc.es](mailto:anna.abbadessa@usc.es)

ORCID: 0000-0003-3095-4086


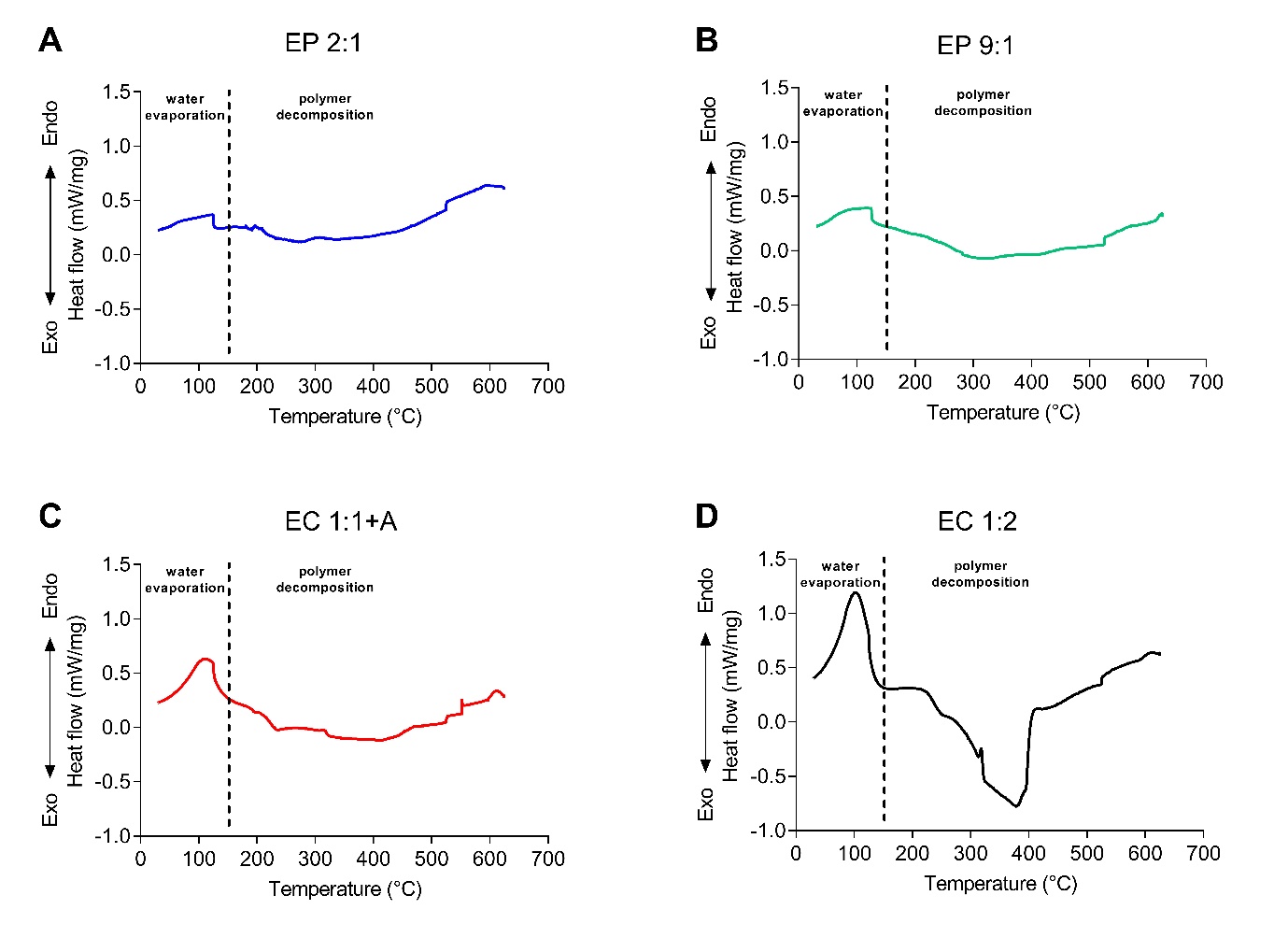


**Figure S1.** DSC curves over the entire temperature ramp (30-625°C) for four selected PECs prototypes made of a EH:PEI ratio of 2:1 (EP 2:1 (A)), a EH:PEI ratio of 9:1 (EP 9:1 (B)), a EH:CH:AG ratio of 1:1:2 (EC 1:1+A (C)), and a EH:CH ratio of 1:2 (EC 1:2 (D)).


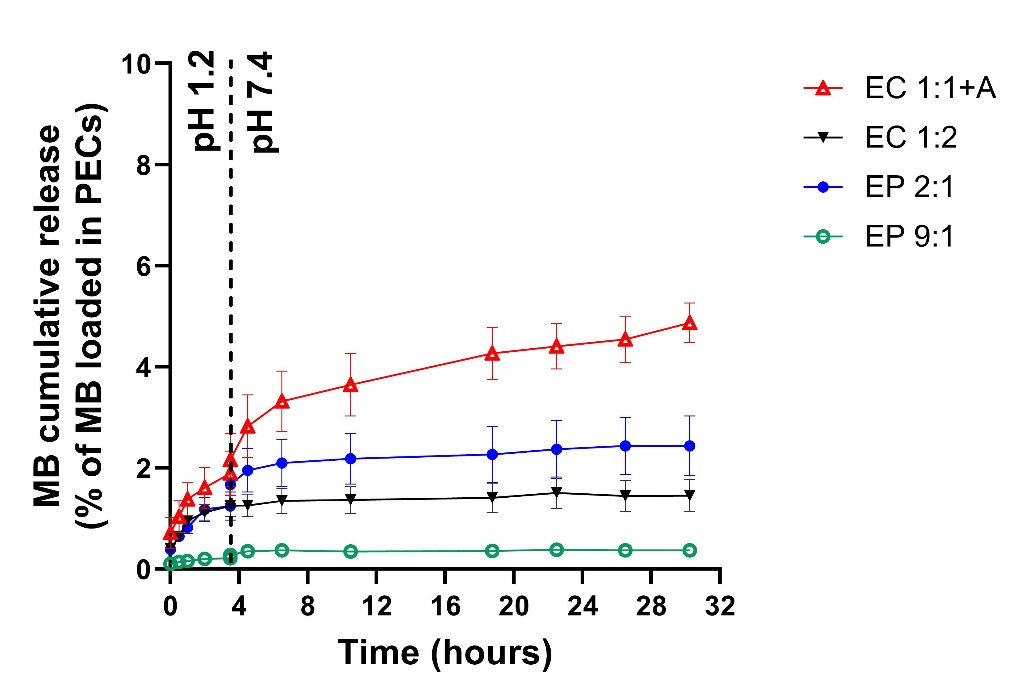


**Figure S2.** Cumulative release of MB expressed as % of MB loaded in PECs.
